# Transcriptomic differences between bleached and unbleached hydrozoan *Millepora complanata* following the 2015-2016 ENSO in the Mexican Caribbean

**DOI:** 10.7717/peerj.14626

**Published:** 2023-01-18

**Authors:** Víctor H. Hernández Elizárraga, Norma Olguín-López, Rosalina Hernández-Matehuala, Juan Caballero-Pérez, César Ibarra-Alvarado, Alejandra Rojas-Molina

**Affiliations:** 1 Posgrado en Ciencias Químico Biológicas, Facultad de Química, Universidad Autónoma de Querétaro, Querétaro, México; 2 European Bioinformatics Institute, EBI-Hinxton, Cambridge, United Kingdom; 3 Laboratorio de Investigación Química y Farmacológica de Productos Naturales, Facultad de Química, Universidad Autónoma de Querétaro, Querétaro, México

**Keywords:** *Millepora complanata*, RNA-seq, Transcriptomics, Coral bleaching, Thermal stress, Hydrocoral, Fire coral

## Abstract

The 2015-2016 El Niño-southern oscillation or “ENSO” caused many *M. complanata* colonies that live in the Mexican Caribbean to experience extensive bleaching. The purpose of this work was to analyze the effect of bleaching on the cellular response of *M. complanata*, employing a transcriptomic approach with RNA-seq. As expected, bleached specimens contained a significantly lower chlorophyll content than unbleached hydrocorals. The presence of algae of the genera *Durusdinium* and *Cladocopium* was only found in tissues of unbleached *M. complanata*, which could be associated to the greater resistance that these colonies exhibited during bleaching. We found that 299 genes were differentially expressed in *M. complanata* bleached colonies following the 2015-2016 ENSO in the Mexican Caribbean. The differential expression analysis of bleached *M. complanata* specimens evidenced enriched terms for functional categories, such as ribosome, RNA polymerase and basal transcription factors, chaperone, oxidoreductase, among others. Our results suggest that the heat-shock response mechanisms displayed by *M. complanata* include: an up-regulation of endogenous antioxidant defenses; a higher expression of heat stress response genes; up-regulation of transcription-related genes, higher expression of genes associated to transport processes, inter alia. This study constitutes the first differential gene expression analysi*s* of the molecular response of a reef-forming hydrozoan during bleaching.

## Introduction

Coral reefs, built by scleractinian corals and hydrocorals, play a substantial role in marine ecology and human sustainability. Reef-forming cnidarians establish a mutualistic symbiosis with photosynthetic algae of the Symbiodiniaceae family (González-Pech et al., 2019). Cnidaria-Symbiodiniaceae symbiosis is essential for coral reef health since photosynthetic algae supply most of the energetic requirements of their cnidarian hosts, which enables coral skeleton growth by calcium carbonate deposition (Cziesielski, Schmidt-Roach & Aranda, 2019).

At the present time, coral reefs are seriously endangered by an accelerated human-induced climate change, which has resulted in a drastic worldwide coral cover decline that seriously threatens marine biodiversity (Hein et al., 2019; Rinkevich, 2019). The current increased and prolonged thermal stress causes a decrease in the population of symbiotic algae within the endodermal cells of the cnidarian, leading to an imbalance of the Cnidaria-Symbiodiniacea symbiosis, which triggers coral bleaching (Hoegh-Guldberg, 1999; Hughes et al., 2017; Lesser, 2006; Suggett & Smith, 2020).

The large-scale climatic phenomenon known as the El Niño-southern oscillation or “ENSO” significantly contributes to coral bleaching events. During a bleaching episode, a state of oxidative stress, generated by the overproduction of reactive oxygen species (ROS), causes the expulsion of algae from the host. These perturbations induced by climate change seriously threaten the integrity and resilience of coral reefs and marine ecosystems that depend on them (Montero-Serra et al., 2019). In recent years, high mortality rates of coral reefs all over the world have triggered a rapid deterioration of reef structures and a far-reaching environmental impact (Eakin, Sweatman & Brainard, 2019).

Numerous investigations aimed at understanding the etiology and effects of coral bleaching have been conducted on reef-forming cnidarians of the Anthozoa class employing transcriptomics approaches. These studies have demonstrated that thermal stress induces the differential expression of genes encoding different cellular components and diverse molecular processes including oxidative stress, Ca^2+^ homeostasis, cytoskeletal organization, cell death, calcification, metabolism, protein synthesis, heat shock proteins (HSP), immunity, and transposons (DeSalvo et al., 2008; Kenkel, Meyer & Matz, 2013; Anderson et al., 2016; Maor-Landaw et al., 2017; Zhang et al., 2022).

Evidently, whole-transcriptome gene expression studies have allowed a much deeper insight into complexity of the holobiont’s stress response (Mayfield et al., 2014; Kaniewska et al., 2015; González-Pech et al., 2017). Hydrocorals are considered as the second most important coral reef builders, however, the majority of investigations aimed at evaluating the influence of heat stress on the cellular processes of reef forming cnidarians have focused on Anthozoa species, and to date very little is known about the cellular response of Hydrozoa species to thermal stress.

Two previous proteomics studies, carried out by our research group, demonstrated that thermal stress induced changes on the soluble proteome of *Millepora alcicornis* and *M. complanata*. A differential expression of proteins related to exocytosis, calcium homeostasis, cytoskeleton organization, and toxins was detected in *M. alcicornis* (Olguín-López et al., 2019), whereas *M. complanata* showed differential expression of proteins related to primary metabolism, cytoskeleton, signaling, DNA repair, stress response, redox homeostasis, exocytosis, calcium homeostasis, and toxins (Hernández-Elizárraga et al., 2019). Recently, we reported the first transcriptome analysis of the *M. complanata* holobiont (Hernández-Elizárraga et al., 2021), however, no prior transcriptomics studies have addressed the impact of increased ocean temperature on the cellular response of hydrocorals. Therefore, the objective of this study was to analyze the transcriptomic response of *M. complanata* holobionts that were exposed to the 2015-2016 ENSO in the Mexican Caribbean.

## Materials & Methods

### Ethics statements

This investigation was approved by the Research Ethics Committee of the Faculty of Chemistry at the Autonomous University of Querétaro, México (approval code CBQ19/058). The collection of *M. complanata* specimens was authorized by Secretaría del Medio Ambiente y Recursos Naturales de México (SEMARNAT) (permit number PFP-DGOPA-139/15).

### Hydrocorals sampling

Unbleached (UMc) and bleached (BMc) *M. complanata* fragments were collected in the Parque Nacional Arrecife de Puerto Morelos, Quintana Roo, México (21°00′00′ and 20°48′33′ North latitude and 86°53′14.40′ and 86°46′38.94′ West longitude) in November 2016, after the ENSO event of 2015–2016. Overall, twenty samples, each one with an approximate area of 25 cm^2^, were collected from the edges of bleached (BMc) and unbleached (UMc) *M. complanata* colonies located at ∼5 m-depth, and at a distance of at least 10 m between them. Each fragment, either bleached or unbleached, was sampled from different colonies. Collected hydrocoral samples were immediately preserved in liquid nitrogen. Seawater temperature records during November 2016 and previous years were obtained from https://seatemperature.info (data recollection method: satellite-based remote sensing in combination with *in situ* data).

### Quantification of chlorophyll content

For chlorophyll quantification, the method described by Shirur et al. (2014) was followed with some modifications. Chlorophylls from the endosymbiotic algae contained in the tissues of bleached (BMc; *n* = 3) and unbleached (UMc; *n* = 3) hydrocorals were extracted by adding an acetone and dimethyl sulfoxide (95:5 v/v) mixture and incubating for 24 h at −20 °C in the dark. Thereafter, the absorbance of the extracts was measured at 630, and 663 nm, using a Benchmark Plus microplate spectrophotometer (Bio-Rad Laboratories, Hercules, CA, USA). Chlorophyll contents (a and c2) were calculated using the equation of Jeffrey & Humphrey (1975) and expressed as µg/mL. Statistical significance difference between chlorophyll contents of UMc and BMc samples was assessed with a Student’s *t*-test (*p* < 0.05).

### Genomic DNA extraction and Symbiodiniaceae detection by rDNA markers

Genomic DNA was extracted from three UMc and BMc biologically independent *M. complanata* samples with a CTAB (Cetyl Trimethyl Ammonium Bromide) adapted method (Dellacorte, 1994). In brief, hydrocoral fragments were powdered with mortar and pestle in liquid nitrogen. Extractions were carried out with 0.1 g of hydrocoral tissue and one mL of CTAB buffer (CTAB 2%, NaCl 1.4 M, EDTA 0.2 M, and Tris–HCl 0.1 M pH 8.8). Afterward, 500 µL of chloroform-isoamyl alcohol (24:1) were added. Extracted DNA samples were precipitated with a 4 M sodium acetate buffer and resuspended in purified water. Symbiodiniaceae genotypes were determined with specific primers for PCR amplifications of rDNA markers according to the Symbiodiniaceae identification method proposed by Correa, McDonald & Baker (2009) (Mieog et al., 2007). Primers employed for the genera *Symbiodinium* (ITS2 locus), *Cladocopium* (ITS1 locus), *Durusdinium* (ITS1 locus), and *Breviolum* (LSU-28S locus) are listed in Table S1. PCR reactions were performed in 20 µL volumes containing 7.5 µL of sterile water, 2 µL of 10X Taq Buffer with KCl, 1.5 µL MgCl 25 mM, 2 µL of genomic DNA, 2.5 µL of 2.5 M forward and reverse primers, and 0.5 U/µL of Taq polymerase (catalog #AAJ64594XEX; Thermo Fisher Scientific, Waltham, MA, USA ). Reactions were carried out in a T100 Bio-rad thermocycler under the following conditions: step 1, 95 °C during 10 min; step 2, 35 cycles consisting of 95 °C during 30 s, 60 °C during 30 s, and 72 °C during 30 s; step 3, 72 °C during 10 min. Amplification products were resolved in 4% agarose gels for 45 min at 75 V and were sent to the IPICyT (Instituto Potosino de Investigación Científica) for DNA sequencing with a 3,130 Genetic Analyzer by Applied Biosystems (Thermo Fisher Scientific, Waltham, MA, USA). The amplification of rDNA markers was confirmed by alignment of the sequences to the Non-redundant NCBI database (accessed May 29, 2019) using Blastn with an e-value threshold of 1.0E−6. rDNA sequences from the detected Symbiodiniaceae algae were used to perform a phylogenetic analysis by the Neighbor-Joining tree inference method (seeded guide trees and hidden Markov model profile-profile) to explore the relationship of *M. complanata* symbionts and other symbionts, whose rDNA sequences are deposited in the GenBank.

### RNA isolation, sequencing, and metatranscriptome assembly

RNA was isolated from biologically independent UMc (*n* = 3) and BMc (*n* = 5) specimens (0.1 g of tissue per sample) according to a method previously reported (Hernández-Elizárraga et al., 2021). Briefly, hydrocoral tissue samples were mixed with 1 mL of extraction buffer (150 mM Tris base, 2% SDS, 1% 2-mercaptoethanol, and 100 mM EDTA pH 7.5 with saturated boric acid). Then, 100 µL of potassium acetate 5 M, 250 µL of absolute ethanol, and 500 µL of chloroform-isoamyl alcohol (25:1) were added and vortexed for 1 min. Samples were centrifuged at 12,000 rpm for 10 min, afterward, the top layer was transferred to a new tube and 500 µL of LiCl 8 M were added. RNA was precipitated overnight. Extracted RNA was centrifuged at 12,000 rpm for 10 min, washed with 250 µL of ethanol 70% and resuspended in sterile water.

RNA quality was assessed with an Agilent™ Eukaryote Total RNA Nano chip (Mueller, Lightfoot & Schroeder, 2004). cDNA libraries were constructed using the TruSeq RNA Library prep kit as per manufacturer instructions. Subsequently, cDNA libraries (multiplexed) were sequenced on the Illumina NextSeq platform using 2 ×150 bp (four lanes). Raw reads were analyzed with FASTQC for quality control (Brown, Pirrung & McCue, 2017). Raw data were submitted to the Sequence Read Archive (SRA) of the NCBI as Bioproject: PRJNA524427 and Biosample: SAMN11026413. To get a better isoform reconstruction from the RNA-seq data, each cDNA library (per sample) was independently assembled using rnaSPAdes (Bushmanova et al., 2019). Then, a combined base metatranscriptome was made by clustering contig sequences with CD HIT-EST to reduce sequence redundancy (Li & Godzik, 2006; Fu et al., 2012). Assembled metatranscriptome was submitted to the Transcriptome Shotgun Assembly (TSA) repository from the NCBI. Thereafter, Kallisto (Bray et al., 2016) was used to quantify expression levels of each library and an abundance table was created. Additionally, data were grouped into two expression tables comprising effective counts (CPM) and transcripts per million (TPM). Statistics of the assembly were calculated with Python and R custom scripts (the scripts used in this study are found in https://github.com/vhelizarraga/Effect_thermal_strees_on_Mcomplanata.git). The statistical power for this experimental design was calculated with RNASeqPower in R. A power >0.66 was retrieved for a fold-change of two using a 10% probability of a Type 1 error occurring (alpha = 0.1). To explore the variation among unbleached and bleached samples, we carried out a Multidimensional Scaling analysis (MDS) to visualize differences between unbleached and bleached hydrocorals, using CPM data in R and the edgeR package (Robinson, McCarthy & Smyth, 2010).

### Functional annotation, gene ontology terms assignment, and KEGG analysis

Sequences were annotated by sequence similarity using the Blastx software against the non-redundant NCBI protein database with an e-value threshold of 1.0E-6 (accessed on 09/01/2018). After functional annotation, Blastx (e-value threshold <1.0E−6) hits were employed to perform the taxonomy analysis with MEGAN *v.* 6.19.8 (https://uni-tuebingen.de/fakultaeten/mathematisch-naturwissenschaftliche-fakultaet/fachbereiche/informatik/lehrstuehle/algorithms-in-bioinformatics/software/megan6/) (Huson et al., 2007). First, the distribution of sequences at Domain rank was determined. Then, Top-20 hit species distributions were retrieved. Transcripts belonging to cnidarian, symbiont, and microbiome were separated using Blastx hits as follows: cnidarian sequences were obtained from Metazoa cores; symbiont contigs were retrieved from Sar supergroup, and sequences from Bacteria, Archaea, and Virus ranks were assigned as microbiome elements. Statistics of separated sequences were obtained with Python and R scripts (https://github.com/vhelizarraga/Effect_thermal_strees_on_Mcomplanata.git). Completeness analysis for split host, symbiont, and microbiome transcriptomes were assessed by comparing the transcripts with sets of the Benchmarking Universal Single-Copy ortholog (BUSCO) *v.* 4.1.2 corresponding to Eukaryota, Metazoa, and Bacteria ortholog sets from the OrthoDB v10 database (Simão et al., 2015). Finally, Blastx hits were employed to retrieve gene ontology terms using Blast2GO (Conesa et al., 2005) for the following sub-ontology groups: molecular function (MF), biological process (BP), and cellular component (CC). Significant gene number differences between bleached and unbleached samples were calculated with the Chi-square test. Finally, the pathways from the Kyoto Encyclopedia of Genes and Genomes (KEGG) database were retrieved for UMc and BMc with Blast2GO (Conesa et al., 2005).

### Differential gene expression analysis

To detect the transcriptomic differences between bleached and unbleached *Millepora complanata* holobionts following the 2015-2016 ENSO in the Mexican Caribbean, a pairwise differential expression analysis was carried out using edgeR (Robinson, McCarthy & Smyth, 2010) for BMc. Briefly, CPM values from the data sets were calculated and samples were filtered using a CPM value of 3 and thereafter, they were normalized using the TMM method (Robinson, McCarthy & Smyth, 2010). Then, a quasi-likelihood ratio test (GLM) was employed and the number of differentially expressed genes (DEGs) was retrieved using a false discovery rate (FDR) <0.05. An enrichment analysis, based on hypergeometric distribution followed by FDR correction, and a gene network analysis were carried out for DEGs from bleached samples to describe the relationship between enriched pathways detected for bleached samples, using ShinyGO v0.66 (Ge, Jung & Yao, 2020).

### Validation of DEGs by semi-quantitative PCR

Biologically independent samples of unbleached (*n* = 3) and bleached (*n* = 3) *M. complanata* were employed for gene expression validation. Five DEGs were randomly selected, and a semi-quantitative PCR analysis of these genes was performed. Primers for PCR amplifications targeting myosin heavy chain, superoxide dismutase, 10 kDa heat shock protein, zinc-metalloproteinase, and voltage-dependent L-type calcium channel subunit beta-2 were designed with Primer3Plus (Table S2). PCR reactions were performed in 20 µL (7.5 µL of sterile water, 2 µL of 10X Taq Buffer with KCl, 1.5 µL MgCl 25 mM, 2 µL of cDNA, 2.5 µL of 2.5 M forward and reverse primers, and 0.5 U/µL of Taq polymerase (catalog #AAJ64594XEX; Thermo Fisher Scientific, Waltham, MA, USA). The housekeeping gene encoding S′adenosyl-l-methionine (SAM) synthase was used as an expression level control. Each amplification was carried out in a T100 Bio-rad thermocycler under the following conditions: a) 95 °C during 10 min; b) 35 cycles consisting of 95 °C during 30 s, 60 °C during 30 s, and 72 °C during 30 s; and c) 72 °C during 10 min. Amplification products were resolved in 4% agarose gels for 1 h at 75 V. Images from electrophoresis gels were obtained with a ChemidocMP (Bio-Rad, Hercules, CA, USA) equipment and processed with the ImageLab software v6.0.1 (Bio-Rad, Hercules, CA, USA). Briefly, intensity of the signals (*n* = 3 for each gene obtained from unbleached and bleached hydrocoral samples) were filtered, and analyzed using Quantity Tools. The normalized data, expressed as a function of pixel density (AU unities) were used to determine the statistical significance of relative expression between selected DEGs with a Student’s *t*-test (*p* < 0.05).

## Results

### Degree of bleaching and Symbiodiniaceae genotypes

Fragments from unbleached (UMc) and bleached (BMc) *Millepora complanata* specimens were collected in the Mexican Caribbean after the 2015-2016 ENSO event in November 2016. According to data recorded by the NOAA’s coral-reefs watch satellite monitoring, a level 1 alert was experienced in the Mexican Caribbean from August to November 2016 with a 60% probability of bleaching due to thermal stress. (Source:  https://coralreefwatch.noaa.gov/index.php). The average seawater temperature in Puerto Morelos, Quintana Roo, Mexico during November 2016 was higher than that observed in the years 2007, 2008, 2010, 2011, 2012, and 2014 (Fig. S1) (Source: https://seatemperature.info).

**Figure 1:**
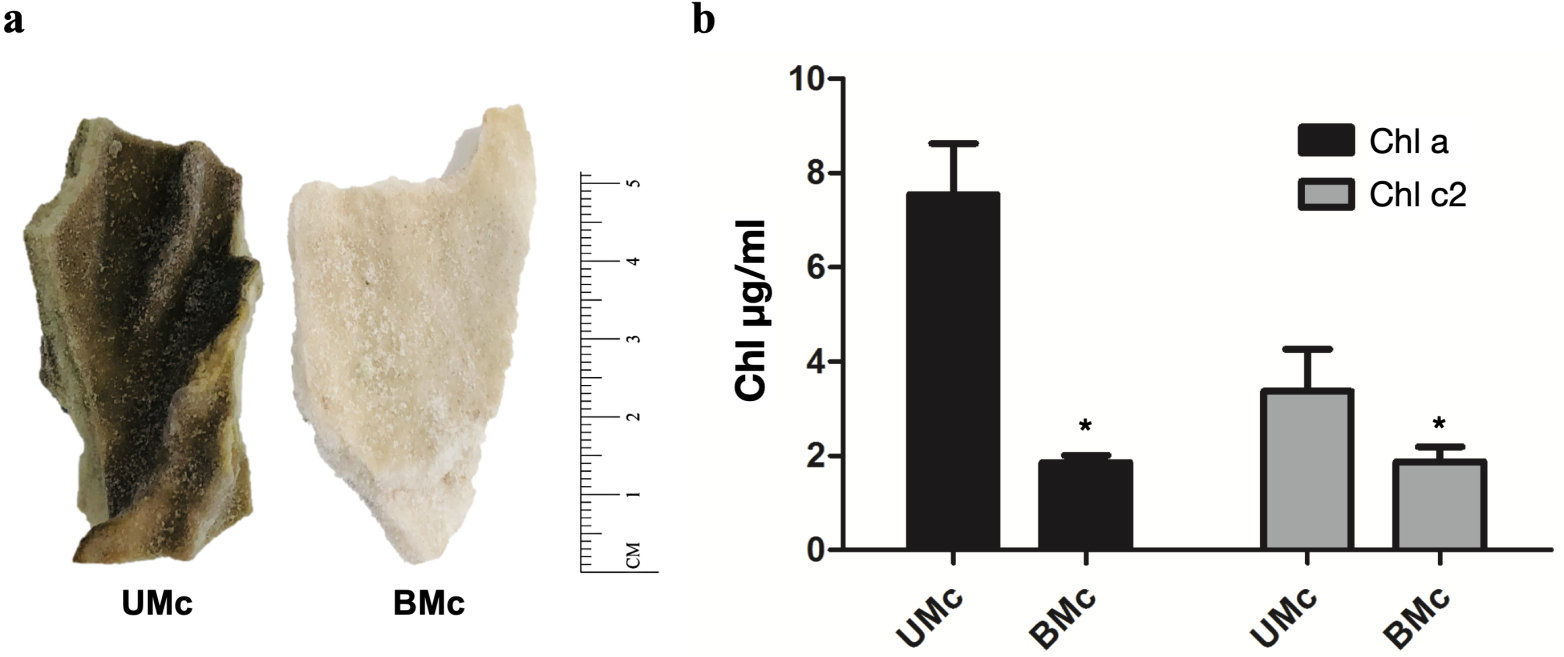
Sample collection and quantification of chlorophyll. (A) Representative fragments of *M. complanata* specimens collected in the Mexican Caribbean. UMC, Unbleached *M. complanata.* BMc, Bleached *M. complanata.* (B) Quantification of chlorophyll a and c2 content from UMc (*n* = 3) and BMc (*n* = 5) specimens.

Representative *M. complanata* fragments (∼5 cm length) are shown in Fig. 1A. BMc specimens were almost colorless compared to UMc samples. The chlorophyll content in *M. complanata* tissues was measured to determine the degree of bleaching of hydrocoral colonies. As expected, BMc samples showed a significantly lower (*p* < 0.05) chlorophyll a and c2 contents (1.86 ± 0.26 µg/mL and 1.88 ± 0.55 µg/mL, respectively) than that of UMc samples (7.54 ± 1.88 µg/mL and 3.37 ± 1.55 µg/mL) (Fig. 1B). The Symbiodiniaceae genotypes identified based on the rDNA markers (best hits) are presented in Table 1. UMc specimens showed the presence of four Symbiodiniaceae genera: *Symbiodinium* spp. (MH612580.1), *Breviolum* spp. (DQ200698.1), *Cladocopium* spp. (AF360576.1), and *Durusdinium* spp. (JQ516983.1), while only *Symbiodinium* spp. (MH612580.1) and *Breviolum* spp. (DQ200698.1) were found in BMc. A phylogenetic tree showing agglomerative neighbor-joining (bottom-up) for *Symbiodinium* spp., *Breviolum* spp., *Cladocopium* spp., and *Durusdinium* spp. is shown in Fig. S2.

**Table 1:** Symbiodiniaceae identification with Blastn.

**Hit description**	**Scientific name**	**Samples**	**Max score**	**Query cover**	**E value**	**Per. Ident.**	**Hit accession**
*Symbiodinium* sp. isolate CV94 internal transcribed spacer 1	*Symbiodinium* sp. (formerly *Symbiodinium* clade A)	Mc1; Mc2; Mc3; BMc3	268	87%	1E-67	89.72%	MH612580.1
s15b1_p 28S large subunit ribosomal RNA gene	*Breviolum* sp. (formerly *Symbiodinium* type B clone)	Mc1; Mc2; Mc3; BMc5;	187	98%	1E-43	97.30%	DQ200698.1
*Symbiodinium* sp. clade_C 18S ribosomal RNA gene, partial sequence; internal transcribed spacer 1	*Cladocopium* sp. (formerly *Symbiodinium* sp. clade C	Mc1; Mc2; Mc3	45	51%	1E-17	89.47%	AF360576.1
Uncultured clade D *Symbiodinium* sp. clone 0907_ZHM3_5	*Durisdinium* sp. (formerly *Symbiodinium* sp. clade D)	Mc1; Mc2; Mc3	78.7	59%	4E-11	97.78%	JQ516983.1

### General description of the metatranscriptome

General analysis from *De novo* assembly of *M. complanata*’s base metatranscriptome containing both UMc and BMc transcripts resulted in 412 660 contigs. These sequences possessed an average size of 412 bp, a maximal assembled contig of 37 774 bp, and a GC content of 43.0%. The assembled holobiont metatranscriptome was deposited in GenBank under the accession GIXI00000000. From the metatranscriptome 169, 236 sequences were annotated by sequence similarity using the non-redundant NCBI database. After sequence separation, summarized benchmarks in BUSCO notation for host, symbiont, and microbiome subsets were retrieved (Fig. S3A). This analysis showed that 86.9% of the core genes were detected, including complete and partial BUSCOs, for the host database; 56.86% for the symbiont database; and 20.97% of core microbial genes were recovered.

### Contribution of holobiont associated domains to transcriptome composition

Taking into account taxonomy-assigned contigs using MEGAN (Huson et al., 2007) (169, 236 sequences with an e-value <1.0E^−6^)*,* the assembled transcripts corresponded to Eukaryotic (84.9%), Bacteria (14.8%), Archaea (0.2%), and Virus (0.2%) sequences. Hit sequences were classified as follows: cnidarian sequences (37.8%), symbiont sequences (37.4%), and sequences from the microbiome (15.2%) (Fig. S3B). In addition, top-20 hit by species (based on the number of sequences matching species) showed that *M. complanata* metatranscriptome mainly contained putative homologs to *Symbiodinium microadriaticum* and *Hydra vulgaris* (Fig. S4)*.* Summary statistics of sequences before and after splitting is shown in Table 2.

Employing Blast2GO, gene ontology terms were assigned to cnidarian, symbiont, and microbiome subsets under unbleached and bleached conditions. Figure S5 displays the GO terms across BP, CC, and MF sub-ontologies (left panel) and significant terms, based on gene numbers per subset under unbleached and bleached conditions (right panel). Metabolic process, membrane, and catalytic activity were the most represented terms for cnidarian, symbiont, and microbiome subsets, respectively.

Annotations were subjected to the KEGG pathway analysis for unbleached and bleached samples. The most represented metabolic pathways (based on the number of sequences per pathway) for UMc and BMc are shown in Fig. S6. We found that 147 KEGG pathways were retrieved from the *M. complanata* holobiont. Regarding bleached samples, pathways comprising the highest number of gene products included: thiamine metabolism (map00730), sucrose metabolism (map00500), and arginine and proline metabolism (map00330), among others.

**Table 2:** Summary statistics of the sequences before and after splitting.

**Holobiont sequences (Metaorganism)**	
Number of total paired-end reads	181, 710, 932
Number of filtered and assembled contigs	412, 660
Maximum contig length	37, 774 bp
Mean contig length	412.3 bp
Mean GC content	43.0%
Taxonomy-assigned counts	169, 236
**Cnidarian sequences** **(** ***Millepora)***	
Number of contigs	64, 077
Maximum contig length	37, 774 bp
Mean contig length	616.2 bp
Mean GC content	40.9%
**Symbiont sequences (Symbiodiniaceae)**	
Number of contigs	63, 400
Maximum contig length	10, 204 bp
Mean contig length	499.7 bp
Mean GC content	51.1%
**Microbiome sequences**	
Number of contigs	25, 561
Maximum contig length	8,965 bp
Mean contig length	382.9 bp
Mean GC content	51.9%

### Transcriptomic differences between bleached and unbleached hydrocorals

**Figure 2:**
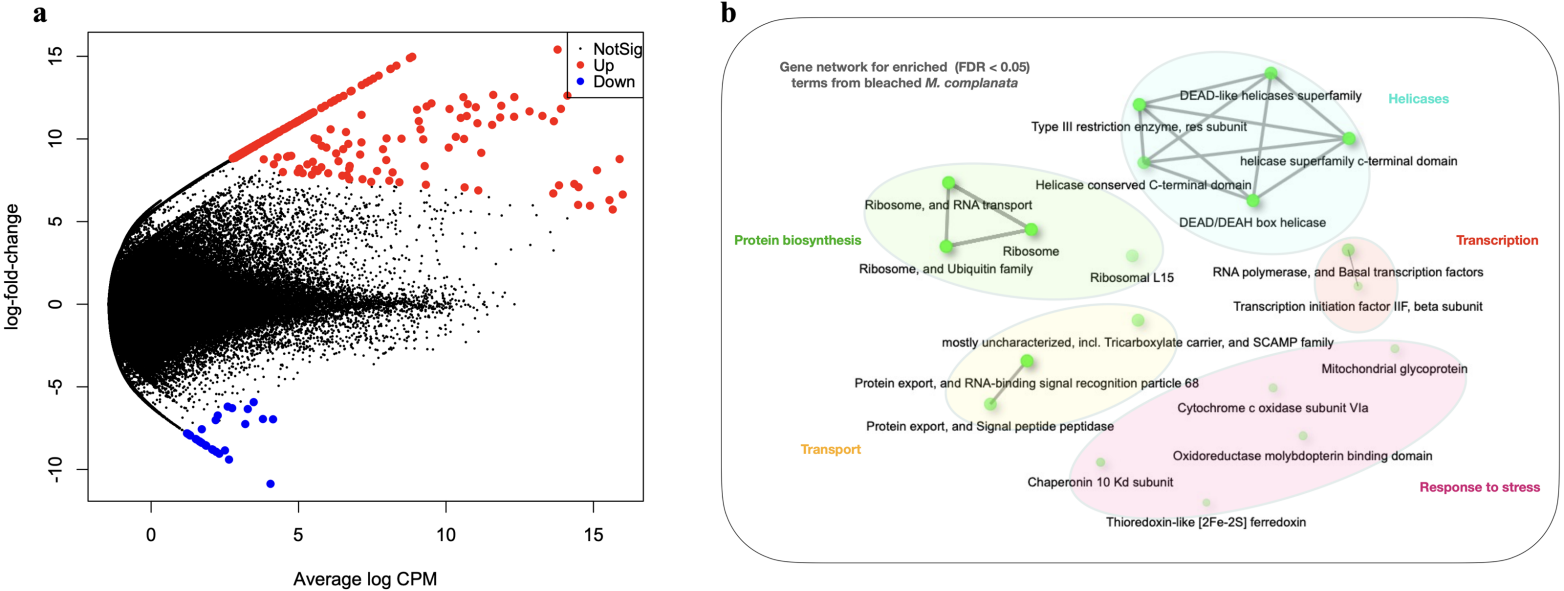
Differential gene expression analyses. (A) Volcano plot showing differentially expressed genes (DEGs) in bleached *M. complanata* determined with edgeR. (B) Gene network displaying enriched gene ontology terms from DEGs in bleached *M. complanata*. Two pathways (nodes) are connected if they share 20% or more genes. Darker nodes are more significantly enriched gene sets. Bigger nodes represent larger gene sets. Thicker edges represent more overlapped genes.

The arrangement of points derived from the MDS analysis indicated a better clustering for unbleached samples than that obtained for bleached specimens (Fig. S7). Genes whose expression was modified in bleached *M. complanata* were determined. Pairwise expression analysis (*p* < 0.05) resulted in 299 significantly differentially expressed genes, from which 265 were up-regulated and 34 were down-regulated in BMc (Fig. 2A). Annotated DEGs are shown in Table S3. We selected a group of relevant differentially expressed genes identified for bleached specimens, which are shown in the Table 3. Enrichment analysis based on hypergeometric distribution followed by FDR correction for DEGs showed overrepresented functional categories in bleached samples (*p*-value cutoff of 0.05), which included ribosome, RNA polymerase, chaperone, oxidoreductase, and basal transcription factors, among others. Gene networks explaining the relationship between enriched GO terms detected in BMc are presented in Fig. 2B. Networks represent the hierarchical clustering summarizing the correlation between enriched pathways including helicases, protein biosynthesis, transcription, transport, and response to stress (Fig. 2). The relative expression of five randomly selected DEGs (myosin heavy chain, superoxide dismutase, 10 kDa heat shock protein, zinc-metalloproteinase, and voltage-dependent L-type calcium channel subunit beta-2) was validated using semi-quantitative PCR. The expression levels of all the DEGs subjected to validation were consistent with the pairwise expression analysis (Fig. S8).

## Discussion

### Symbiont diversity

Although hydrocorals are considered the second most important reef builders (Lewis, 2006), they have been poorly studied. Particularly, the effect of climate change on *Millepora* species has not been comprehensively addressed. Here, we describe for the first time, differences observed at the transcriptomic level between unbleached and bleached *M. complanata* holobionts that were exposed to the 2015-2016 ENSO in the Mexican Caribbean, which caused an exceptional rise in the ocean temperature that resulted in massive episodes of coral bleaching worldwide (Banon et al., 2018; Rioja-Nieto & Álvarez Filip, 2019; Romero-Torres et al., 2020).

*M. complanata* has been recognized as a very sensitive species to temperature stress, according to records of bleaching and mortality observed during 1987, 1993, 1995, 1998, 2003, and 2005 (McClanahan et al., 2009). In the present study, we demonstrated that bleached *M. complanata* specimens showed a significant reduction in total chlorophyll content due to the loss of symbionts (Fig. 1B). Interestingly, despite the high temperatures recorded in November 2016 (Fig. S1), some hydrocoral samples, collected simultaneously from colonies located at the same zone and depth in the Mexican Caribbean did not experience bleaching (Fig. 1A), which indicates intra-species variation in *M. complanata* bleaching tolerance. A known mechanism of bleaching resistance is linked to the diversity and abundance of Symbiodiniaceae algae (Swain et al., 2018). It has been demonstrated in Anthozoa species that the presence of heat-tolerant Symbiodiniaceae genera (*e.g.*, *Durusdinium* and *Cladocopium*) improves the resistance to coral bleaching (Qin et al., 2019; Chen et al., 2020; Poquita-Du et al., 2020). However, a high variation related to thermotolerance can be observed within species of the same genera. Even though *M. complanata* has been recognized as a vulnerable species to thermal stress and bleaching, the susceptibility of Symbiodiniaceae species hosted by this hydrocoral has not been previously described. The occurrence of *Symbiodinium* spp. and *Breviolum* spp. was expected (samples Mc1, Mc2, Mc3, BMc3, and BMc5), since previous studies indicated that *M. complanata* from Mexico, Belize, Barbados, and Colombia harbors these Symbiodiniaceae genera (LaJeunesse, 2002; Banaszak et al., 2006; Finney et al., 2010; Grajales & Sanchez, 2016). However, endosymbionts from the genera *Cladocopium* and *Durusdinium* had not been previously identified in *M. complanata* from the Caribbean Sea. Former research has shown that endosymbiont dominance strongly depends on the geographic location (LaJeunesse, 2002; Finney et al., 2010; Grajales & Sanchez, 2016). For example, *Breviolum* spp. is a dominant endosymbiont in *M. complanata* and *M. alcicornis* living in coral reefs of The Bahamas, whereas *Symbiodinium* spp. endosymbionts are prevalent in hydrocorals found in the Belize Barrier Reef (Samayoa et al., 2017). Moreover, it has been demonstrated that depth influences the diversity in Symbiodiniaceae hosted by *M. complanata* (Finney et al., 2010). Evidently, Symbiodiniaceae diversity in hydrocorals depends on the reef habitat, as in the case of reef-forming cnidarians of the Anthozoa class. In the present study, the presence of *Durusdinium* spp. and *Cladocopium* spp. in unbleached *M. complanata* tissues could be associated with the greater resistance that these hydrocorals showed to bleaching, as it has been observed in Anthozoa corals (Pettay & Lajeunesse, 2009; Stat & Gates, 2011) nevertheless, this hypothesis needs to be proved.

**Table 3:** Key differentially expressed genes involved in the putative molecular response of bleached *M. complanata*.

**Sequence identifier**	**Accession**	**Description**	**E-value**	**#GO IDs**	**Expression**	**Fold-change**	**LogFold-change**
Transcription							
Mcom_29474	XP_002160344.2	General transcription factor IIF subunit 2	2.80607E−67	10	U	294.821713453449	8.20369897179935
Mcom_63	XP_002163509.2	DNA-directed RNA polymerase II subunit RPB2	1.5809E−118	24	U	676.546394946371	9.40204506183902
Protein biosynthesis							
Mcom_34105	XP_004206204.1	40S ribosomal protein S30	1.9916E−59	11	U	91.4968770059024	6.51565059669947
Mcom_5558	XP_012554015.1	40S ribosomal protein S14	1.45918E−95	16	U	821.897010849412	9.68281381574302
Mcom_352275	XP_002162844.1	60S ribosomal protein L15	4.53887E−94	5	U	13295.477543909	13.6986479760648
Heat stress response							
Mcom_5556	XP_002162621.1	10 kDa heat shock protein*	3.44982E−33	10	U	123.871181924548	6.95269677975229
Redox homeostasis							
Mcom_162778	JQ994220.1	Superoxide dismutase*	3.67142E−08	6	U	349.582068315757	8.44948737399706
Cytoskeleton							
Mcom_375516	ACY74447.1	Actin	0.0	3	U	302.06468295438	8.23871370550206
Mcom_340435	XP_002160112.1	Radixin 2438	0.0	63	U	64.5557253623457	6.01247314681906
Mcom_289194	Q05000.1	Myosin heavy chain*	4.08753E−78	27	D	147.190539369995	7.20154113533664
Transport							
Mcom_40833	ABD59026.1	Voltage-dependent L-type calcium channel subunit beta-28^*^	1.86888E−139	28	U	296.930773517945	8.21398280987948

**Notes.**

* Expression trend validated by semi-quantitative PCR.

### Contribution of the host, symbiont, and microorganisms to the assembled contigs

Orthologous groups of BUSCO cores were detected for host, symbiont, and microbiome from the *M.complanata* holobiont. Contig-based taxonomic classification showed an approximate hydrocoral-symbiont-microbiome ratio of 2:2:1 (Fig. S3B). These results suggest that *Millepora* holobionts mainly comprise cnidarians and Symbiodiniaceae components*,* while the microbiome represents a smaller fraction of the holobiont’s population. A similar host/symbiont composition has been observed for scleractinian corals, such as *Porites australiensis* (Shinzato, Inoue & Kusakabe, 2014). There is growing evidence demonstrating the important contribution of the microbiome to the coral holobiont survival and performance (Sogin et al., 2017; Osman et al., 2020). Here, we present the first report of the microbial contribution to the *M. complanata* holobiont, however, considering the low representation of bacteria sequences found in the present investigation, future RNA-seq research targeting procaryote RNA is needed in order to improve the characterization of the microbiome of the *Millepora* spp. holobiont. A subsequent KEGG pathway analysis for BMc evidenced that gene products from thiamine metabolism (map00730), sucrose metabolism (map00500), and arginine and proline metabolism (map00330) were the most prevalent pathways. These results represent the first snapshot of the metabolic processes occurring within the *M. complanata* holobiont during bleaching.

### Transcriptomic differences between bleached and unbleached *M. complanata* holobionts

**Figure 3:**
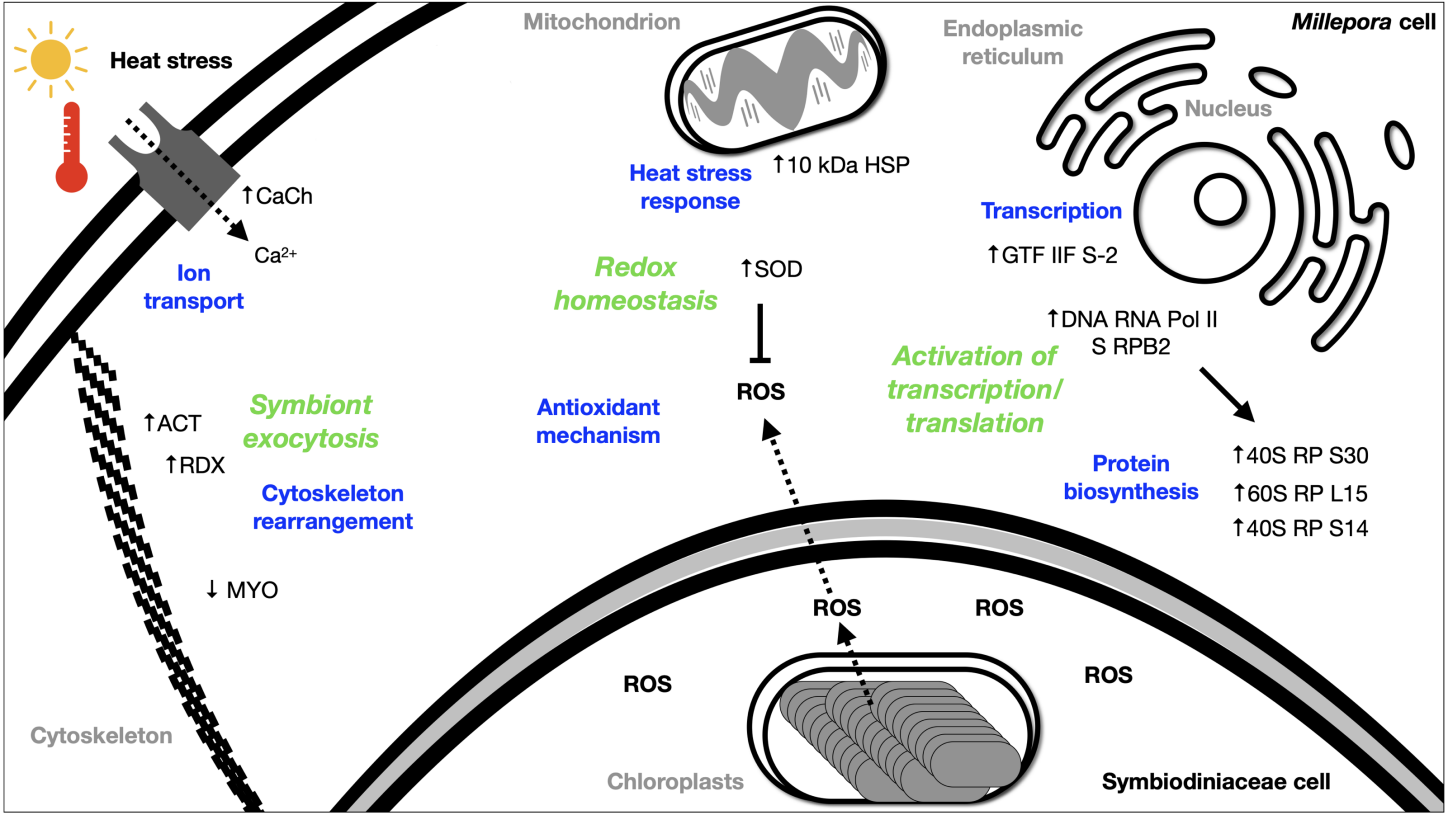
Summary of the putative response mechanism of *M. complanata* in the face of coral bleaching. Acronyms: reactive oxygen species (ROS), 40S ribosomal protein S30 (40S RP S30), 60S ribosomal protein L15 (60S RP L15), 40S ribosomal protein S14 (40S RP S14), general transcription factor IIF subunit 2 (GTF IIF S-2), DNA-directed RNA polymerase II subunit RPB2 (DNA RNA Pol II S RPB2), 10 kDa heat shock protein (10 kDa HSP), Superoxide dismutase (SOD), Voltage-dependent L-type calcium channel subunit beta-2 (CaCh), actin (ACT), radixin 2438 (RDX), Myosin heavy chain (MYO).

The DEGs analysis revealed significant (FDR <0.05) changes in the expression of 299 genes in BMc specimens, which had experienced bleaching during the 2015-2016 ENSO (Fig. 2A). Enriched functional terms (*p*-value cutoff of 0.05) from DEGs included: ribosome, chaperone, oxidoreductase, helicase, RNA polymerase, among others (Fig. 2B). Representative enriched categories for bleached samples and some relevant DEGs (Table 3) are discussed below. A graphical model of the putative molecular response mechanisms displayed by the holobiont *M. complanata*, based on the differentially expressed genes from bleached specimens is presented in Fig. 3.

### Transcription

Several transcriptomic studies have proved that transcriptional regulation is directly affected during coral bleaching (DeSalvo et al., 2008; DeSalvo et al., 2010; Rosic et al., 2011; Souter et al., 2011). In fact, transcriptional modules related to important cellular functions and activities (sequence-specific DNA binding, motor activity, and extracellular matrix structure) have been recognized as significantly correlated with bleaching (Thomas & Palumbi, 2017). Our results showed that RNA polymerase and basal transcription factors were enriched terms (*p*-value cutoff of 0.05) in bleached samples (Fig. 2B). Particularly, some genes like the general transcription factor IIF subunit 2, and the DNA-directed RNA polymerase II subunit RPB2 were up-regulated (Table 3), and no genes related to these processes were down-regulated. These results indicated that bleached hydrocorals did not show a decrease in their transcriptional response (Fig. 3), unlike what has been detected in bleached corals of the Anthozoa class, in which a reduction in transcription and protein biosynthesis has been observed (Mohamed et al., 2016). These findings could imply that there is a different response to thermal stress between organisms of the Anthozoa class and reef-building hydrozoans.

### Protein biosynthesis

The present study provides evidence that BMc samples showed significant changes associated to protein biosynthesis (Fig. 3). Ribosome-related was one of the enriched terms (*p*-value cutoff of 0.05) in BMc (Fig. 2B). In fact, the majority of differentially expressed genes were associated with protein biosynthesis. For example, 40S ribosomal protein S30, 60S ribosomal protein L15, and 40S ribosomal protein S14, were up-regulated (Table 3). In contrast, in the case of some organisms of the Anthozoa class, a decrease in the expression of genes related to protein biosynthesis has been observed as part of an immediate response to heat stress (Voolstra et al., 2009; Pinzón et al., 2015). The detection of a large number of up-regulated genes coding for ribosome constituents in bleached hydrocorals suggests that an uninterrupted protein synthesis is occurring, similarly to what has been previously demonstrated in *M. faveolata* subjected to thermal stress (DeSalvo et al., 2008).

### Cellular transport

Transport-related genes were up-regulated in bleached *M. complanata* hydrocorals (Table S3). In a particular way, the voltage-dependent L-type calcium channel subunit beta-2 gene showed augmented expression levels (Table 3)*.* This finding is in agreement with a previous study carried out in *Pocillopora damicornis* subjected to thermal stress, which showed an up-regulation of ion transporters, such as voltage-gated calcium channels (Crowder et al., 2017). It has been observed that Ca^2+^-channels participate in the calcification process of some scleractinian species. Some voltage-gated Ca^2+^-channels have been directly associated to transepithelial calcium transport and calcium carbonate formation (Zoccola et al., 1999). Previous investigations provided evidence that blockade of voltage-gated Ca^2+^-channel Ca_V_1 significantly diminished calcification in *Stylophora pistillata* and *Galaxea fascicularis* (Marshall, 1996; Tanbutté et al., 1996). Therefore, it is likely that the up-regulation of genes associated with Ca^2+^ transport and signaling in BMc specimens is related to the optimization of cellular transport pathways, like those involved in regulating ion transport and calcium carbonate deposition during thermal stress and bleaching (Fig. 3).

### Redox homeostasis and thermal stress response

It has been widely demonstrated that antioxidant enzymes constitute the first line of defense of reef-forming anthozoans against thermal stress and UV radiation. Particularly, superoxide dismutase, catalase, and ascorbate peroxidase play a key role in both, host and symbiont antioxidant response during bleaching (Lesser et al., 1990; Lesser, 1997; Downs et al., 2002; Lesser, 2011; Hawkins et al., 2015; Krueger et al., 2015; Oakley & Davy, 2018). These enzymes importantly contribute to the maintenance of cnidarian-Symbiodiniaceae homeostasis by neutralizing ROS to counteract oxidative damage (Szabó, Larkum & Vass, 2020). Investigations conducted on *Acropora millepora* and *Montipora digitata* indicated that thermal stress induced an augmented expression of antioxidant enzymes (Souter et al., 2011; Krueger et al., 2015).

In this study, oxidoreductases (*p*-value cutoff of 0.05) were enriched (Fig. 2B) and we observed an up-regulation of the antioxidant enzyme superoxide dismutase in *M. complanata* specimens that had experienced bleaching (Table 3). This stress response is similar to that of Anthozoa species (Downs et al., 2002). In addition to augmented expression of antioxidant enzymes, an up-regulation of HSPs has been considered a ubiquitous molecular response of scleractinian corals to heat stress (Rosic et al., 2011). HSPs are responsible for maintaining critical cellular functions, including protein transport, degradation, aggregation, unfolding, and folding under stress conditions (Sørensen, Kristensen & Loeschcke, 2003). Furthermore, these chaperone proteins play a crucial role in cellular protection against damage caused by temperature stress (Black, Voellmy & Szmant, 1995; Hayes & King, 1995; Fang, Huang & Lin, 1997). In this study, we found that the expression of a 10 kDa-heat shock protein was up-regulated (*p*-value cutoff of 0.05) in BMc (Table 3). Recently, Seveso et al. demonstrated that the coral species *Goniopora lobata, Porites lobata, Seriatopora hystrix*, and *Stylophora pistillata* showed a significant species-specific modulation of HSPs expression in response to bleaching (Seveso et al., 2020). Moreover, one HSP70 (PdHSP70), whose expression was induced by high temperature, has been considered an essential element in the prevention of bleaching of the stony coral *Pocillopora damicornis* (Zhang et al., 2018). Up-regulation of genes related to both antioxidant and heat shock response in bleached *M. complanata* is indicative of the responsive molecular mechanism of these hydrocorals, which may involve their self-defense against the damage caused by thermal stress by activation of redox homeostasis and thermal stress response pathways (Fig. 3). Superoxide dismutase and 10 kDa-heat shock protein could represent important biochemical markers of heat stress in species of the genus *Millepora*.

### Cytoskeleton rearrangement

The cellular mechanisms underlying loss of symbionts following thermal stress involve physiological responses, such as exocytosis or host cell detachment (Weis, 2008). The cytoskeletal rearrangement or cell adhesion disruption occur due to thermal stress (DeSalvo et al., 2008). Considering that bleaching involves exocytosis of symbionts as a result of high temperature, the differential expression of several elements from the host cytoskeleton has been detected in Anthozoa species. For example, five genes related to the actin cytoskeleton were modified by the effect of thermal stress in *M. faveolata* (DeSalvo et al., 2008). In addition, collagen, the major component of the extracellular matrix, and actin an important cytoskeleton protein showed a trend towards elevated expression in bleached *Porites astreoides* (Kenkel, Meyer & Matz, 2013)*.* Distinct patterns of expression of cytoskeletal proteins have been observed in reef-forming cnidarians exposed to thermal stress. In fact, the same cytoskeletal components (*e.g.*, actin, myosin, inter alia) showed both, lower and upper expression after bleaching, depending on the cnidarian species. Regarding *M. complanata*, we found that bleached specimens exhibited modifications in the regulation of genes encoding cytoskeletal proteins (Table S3) including actin, radixin, and myosin (Table 3). These changes could be explained due either to a disruption or a rearrangement of *M. complanata*’s cytoskeletal components related to the release of symbionts during the thermal stress experienced by these hydrocorals in the Mexican Caribbean (Fig. 3).

### Future of bleaching studies in *Millepora* species

Although *Millepora* species are among the most severely affected reef-building organisms by anthropogenic environmental disturbances, up to now very little is known about the cellular mechanisms by which these organisms cope with the stress induced by increased ocean temperature and ultraviolet radiation. Our findings suggest that, even in the absence of Symbiodiniaceae algae, hydrocorals are capable of carrying out essential biochemical and molecular mechanisms, including ribosome, RNA polymerase and basal transcription factors, chaperone, oxidoreductase, transport, among others (Fig. 3).

This is a pioneering study about the transcriptional response of hydrozoan species during heat bleaching, which will contribute to future research aimed at gaining deeper insight into the overall cellular response mechanisms of reef forming hydrozoans and their adaptation and acclimatization strategies against disturbances such as climate change.

##  Supplemental Information

10.7717/peerj.14626/supp-1 Supplemental Information 1 Average water temperature in Puerto Morelos, Quintana Roo, Mexico in November over a decade The data presented in this graph displays average monthly seawater temperature in Puerto Morelos in November based on historical readings over a period of ten years (from 2007 to 2016). Source: https://seatemperature.info.

10.7717/peerj.14626/supp-2 Supplemental Information 2 Phylogenetic tree showing agglomerative neighbor-joining (bottom- up) for *Symbiodinium* spp., *Breviolum* spp., *Cladocopium* spp., and *Durusdinium* spp. hosted in *M. complanata* and related Symbiodiniaceae species from GenBank (Accessions: MH612580.1, MH612579.1, MH612578.1, MH612577.1, MH612576.1, AF333509.1, DQ200698.1, MH728999.1, MH728998.1, MH728997.1, MH647121.1, MK692539.1, AF360576.1, LK934673.1, AJ291535.1, AJ291529.1, AJ291512.1, LC368857.1, JQ516983.1, JQ516941.1, JQ515858.1)*.*

10.7717/peerj.14626/supp-3 Supplemental Information 3 Annotation of the sequences Overall, 169, 236 sequences were annotated by sequence similarity using the non-redundant NCBI database (Accessed 06/01/2018). (A) BUSCO analysis: 86.9% of the core genes were detected, including complete and partial BUSCOs, for the host database; 56.86% for the symbiont database; and 20.97% of core microbial genes were recovered. (B) *M. complanata* taxonomy-assigned contigs using MEGAN. Transcripts corresponded to Eukaryotic (84.9%), Bacteria (14.8%), Archaea (0.2%), and Virus (0.2%) sequences. Hit sequences were classified as follows: cnidarian sequences (37.8%), symbiont sequences (37.4%), and sequences from the microbiome (15.2%).

10.7717/peerj.14626/supp-4 Supplemental Information 4 Top-20 hit species from *M. complanata* metatranscriptome Based on the number of sequences matching species, *M. complanata* metatranscriptome mainly contained putative homologs to *Symbiodinium microadriaticum* and *Hydra vulgaris.*

10.7717/peerj.14626/supp-5 Supplemental Information 5 Gene ontology terms for (A) host, (B) symbiont, and (C) microbiome subsets Right panel: Gene ontology terms across Biological Process (BP), Cellular Component (CC), and Molecular Function (MF) sub-ontologies under unbleached and bleached conditions. Left panel: GO terms with significant gene number differences (*p* < 0.05).

10.7717/peerj.14626/supp-6 Supplemental Information 6 Raw data chlorophyll content

10.7717/peerj.14626/supp-7 Supplemental Information 7 The most represented metabolic pathways (based on the number of sequences per pathway) detected in the *M. complanata* holobiont Overall, 147 KEGG pathways were retrieved for (A) unbleached and (B) bleached *M. complanata*.

10.7717/peerj.14626/supp-8 Supplemental Information 8 Multidimensional Scaling analysis (simplified PCoA) showing the multivariate variation among unbleached and bleached *M. complanata* samples (UMc and BMc, respectively)

10.7717/peerj.14626/supp-9 Supplemental Information 9 (A) Relative expression of five randomly selected DEGs. SOD) superoxide dismutase, MET) zinc-metalloproteinase, MIO) myosin heavy chain, HSP) 10 kDa heat shock protein, and CCH) voltage-dependent L-type calcium channel subunit beta- Asterisks (*) indicate statistical significance (*p* < 0.05). Calculated *p*-values for expression levels: 0.0175, 0.0237, 0.0237, 0.0033, and 0.0039 for SOD, MET, MIO, HSP, and CCH, respectively. Control) S′adenosyl-l-methionine (SAM). (B) Comparison of gene expression trend by RNA-Seq and semi-quantitative RT-PCR.

10.7717/peerj.14626/supp-10 Supplemental Information 10 Primers employed for Symbiodiniaceae identification using PCR

10.7717/peerj.14626/supp-11 Supplemental Information 11 Primers used for DEGs validation with semi-quantitative PCR

10.7717/peerj.14626/supp-12 Supplemental Information 12 Annotated DEGs by the effect of thermal stress in bleached *M. complanata*

## Additional Information and Declarations

### Field Study Permissions

The following information was supplied relating to field study approvals (i.e., approving body and any reference numbers): This investigation was approved by the Research Ethics Committee of the Faculty of Chemistry at the Autonomous University of Querétaro, México (approval code CBQ19/058). The collection of M. complanata specimens was authorized by Secretari’a del Medio Ambiente y Recursos Naturales de México (SEMARNAT) (permit number PFP-DGOPA-139/15).

### DNA Deposition

The following information was supplied regarding the deposition of DNA sequences: The data is available at GenBank: PRJNA524427.

### Data Availability

The following information was supplied regarding data availability: The data is available at GitHub: https://github.com/vhelizarraga/Effect_thermal_strees_on_Mcomplanata.git; vhelizarraga. (2022). vhelizarraga/Effect_thermal_strees_on_Mcomplanata: Transcriptomic differences between bleached and unbleached hydrozoan Millepora complanata following the 2015-2016 ENSO in the Mexican Caribbean (v_1.01). Zenodo. https://doi.org/10.5281/zenodo.7059028.
