# Supplementary material for: Transcriptomic differences between bleached and unbleached hydrozoan Millepora complanata following the 2015-2016 ENSO in the Mexican Caribbean
Source: PeerJ. 2023 Jan 18;11:e14626. doi: 10.7717/peerj.14626 (PMC9864129; doi:10.7717/peerj.14626)
Supplement: Supplemental Information 1 — The data presented in this graph displays average monthly seawater temperature in Puerto Morelos in November based on historical readings over a period of ten years (from 2007 to 2016). Source: https://seatemperature.info. [file peerj-11-14626-s001.docx]

Transcriptomic differences between bleached and unbleached hydrozoan *Millepora complanata* following the 2015-2016 ENSO in the Mexican Caribbean

Víctor Hugo Hernández-Elizárraga1, Norma Olguín-López1, Rosalina Hernández-Matehuala1, Juan Caballero-Pérez3, César Ibarra-Alvarado2 and Alejandra Rojas-Molina2*

1Posgrado en Ciencias Químico Biológicas, Facultad de Química, Universidad Autónoma de Querétaro, Qro., México.

2Laboratorio de Investigación Química y Farmacológica de Productos Naturales, Facultad de Química, Universidad Autónoma de Querétaro, Qro., México.

3EMBL - EBI, Hixton, United Kingdom.

*Corresponding author: Alejandra Rojas-Molina [rojasa@uaq.mx](mailto:rojasa@uaq.mx)


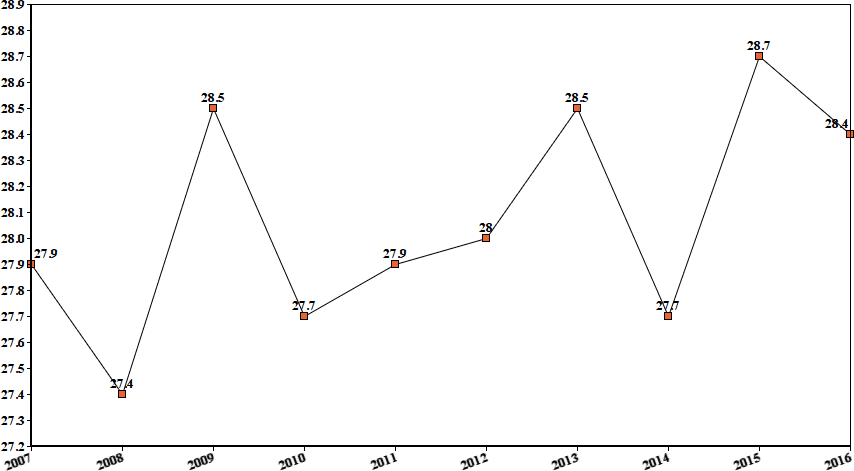


**Supplemental Figure S1.** Average water temperature in Puerto Morelos, Quintana Roo, Mexico in November over a decade. The data presented in this graph displays average monthly seawater temperature in Puerto Morelos in November based on historical readings over a period of ten years (from 2007 to 2016).
