# Supplementary material for: Transcriptomic differences between bleached and unbleached hydrozoan Millepora complanata following the 2015-2016 ENSO in the Mexican Caribbean
Source: PeerJ. 2023 Jan 18;11:e14626. doi: 10.7717/peerj.14626 (PMC9864129; doi:10.7717/peerj.14626)
Supplement: Supplemental Information 2 — (bottom- up) for Symbiodinium spp., Breviolum spp., Cladocopium spp., and Durusdinium spp. hosted in M. complanata and related Symbiodiniaceae species from GenBank (Accessions: MH612580.1, MH612579.1, MH612578.1, MH612577.1, MH612576.1, AF333509.1, DQ200698.1, MH728999.1, MH728998.1, MH728997.1, MH647121.1, MK692539.1, AF360576.1, LK934673.1, AJ291535.1, AJ291529.1, AJ291512.1, LC368857.1, JQ516983.1, JQ516941.1, JQ515858.1). [file peerj-11-14626-s002.docx]

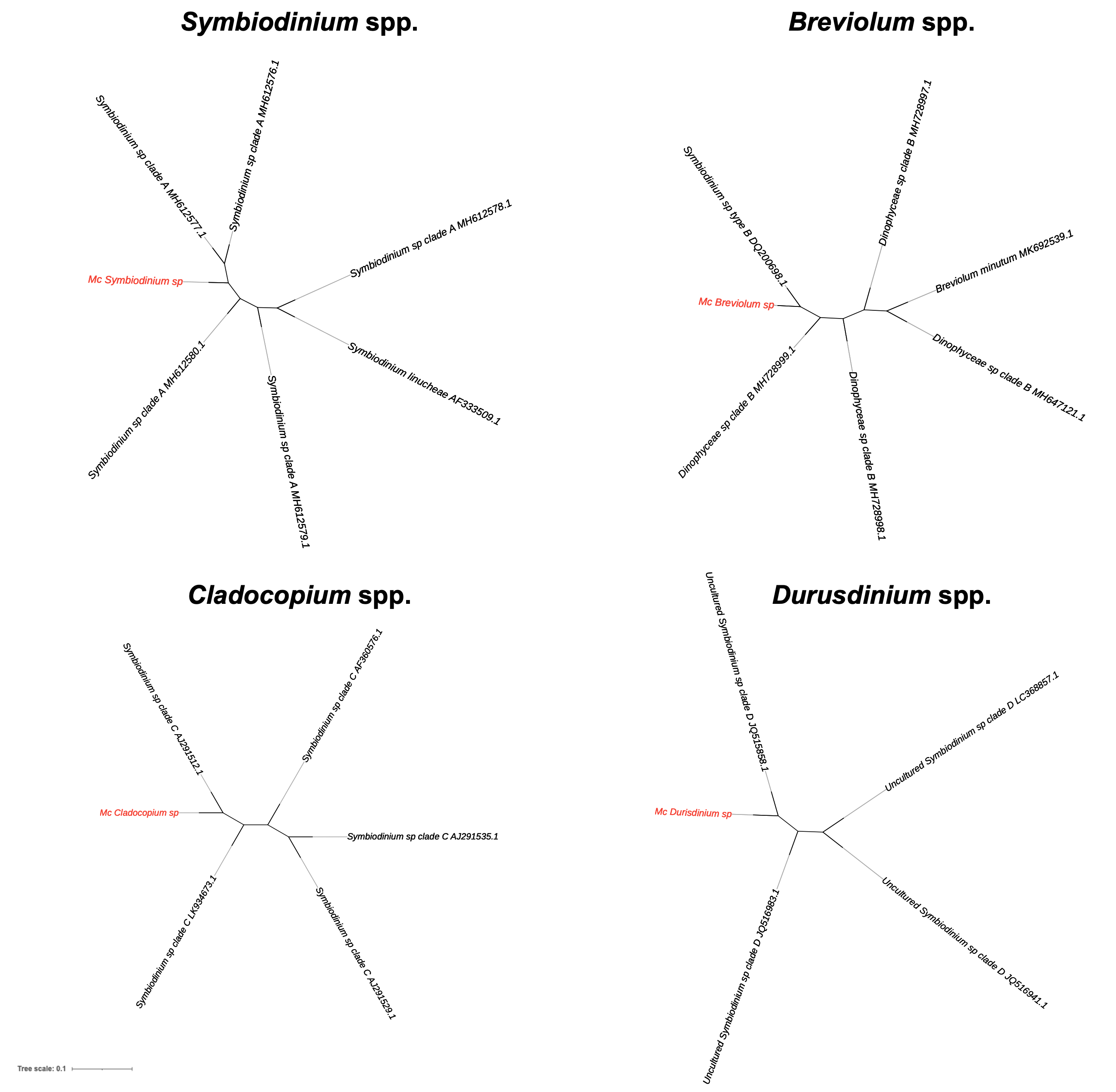


*Mc Cladocopium*

*Mc Durusdinium*

*Mc Breviolum*

*Mc Symbiodinium*

**Supplemental Figure S2.** Phylogenetic tree showing agglomerative neighbor-joining (bottom- up) for *Symbiodinium* spp., *Breviolum* spp., *Cladocopium* spp., and *Durusdinium* spp. hosted in

*M. complanata* and related Symbiodiniaceae species from GenBank (Accessions: MH612580.1, MH612579.1, MH612578.1, MH612577.1, MH612576.1, AF333509.1, DQ200698.1, MH728999.1, MH728998.1, MH728997.1, MH647121.1, MK692539.1, AF360576.1, LK934673.1, AJ291535.1, AJ291529.1, AJ291512.1, LC368857.1, JQ516983.1, JQ516941.1, JQ515858.1)*.*
