# Supplementary material for: Transcriptomic differences between bleached and unbleached hydrozoan Millepora complanata following the 2015-2016 ENSO in the Mexican Caribbean
Source: PeerJ. 2023 Jan 18;11:e14626. doi: 10.7717/peerj.14626 (PMC9864129; doi:10.7717/peerj.14626)
Supplement: Supplemental Information 3 — Overall, 169, 236 sequences were annotated by sequence similarity using the non-redundant NCBI database (Accessed 06/01/2018). (A) BUSCO analysis: 86.9% of the core genes were detected, including complete and partial BUSCOs, for the host database; 56.86% for the symbiont database; and 20.97% of core microbial genes were recovered. (B) M. complanata taxonomy-assigned contigs using MEGAN. Transcripts corresponded to Eukaryotic (84.9%), Bacteria (14.8%), Archaea (0.2%), and Virus (0.2%) sequences. Hit sequences were classified as follows: cnidarian sequences (37.8%), symbiont sequences (37.4%), and sequences from the microbiome (15.2%). [file peerj-11-14626-s003.docx]

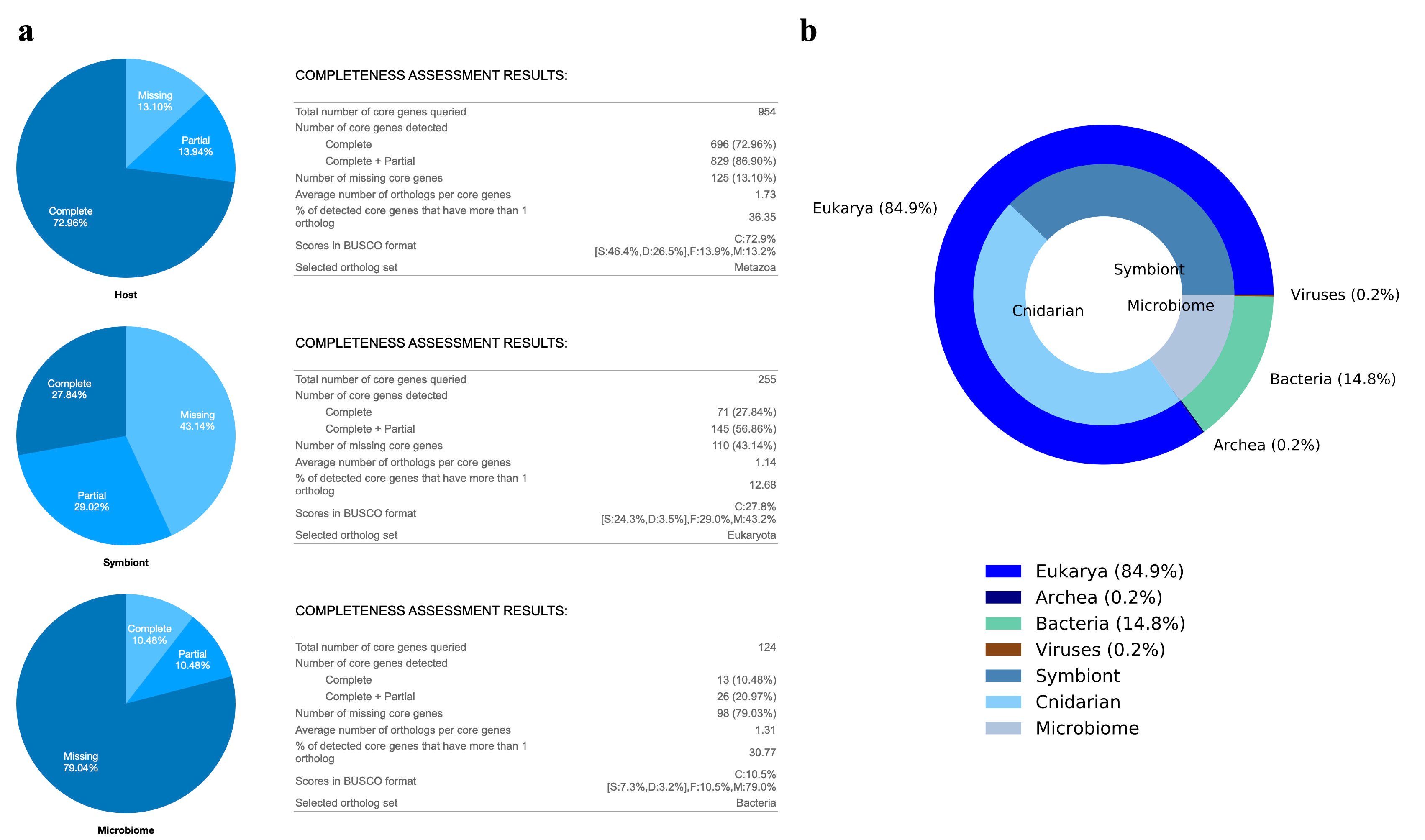


**Supplemental Figure S3.** Annotation of the sequences. Overall, 169, 236 sequences were annotated by sequence similarity using the non-redundant NCBI database (Accessed 06/01/2018). a) BUSCO analysis: 86.9% of the core genes were detected, including complete and partial BUSCOs, for the host database; 56.86% for the symbiont database; and 20.97% of core microbial genes were recovered. b) *M. complanata* taxonomy-assigned contigs using MEGAN. Transcripts corresponded to Eukaryotic (84.9%), Bacteria (14.8%), Archaea (0.2%), and Virus (0.2%) sequences. Hit sequences were classified as follows: cnidarian sequences (37.8%), symbiont sequences (37.4%), and sequences from the microbiome (15.2%).
