# Supplementary material for: Transcriptomic differences between bleached and unbleached hydrozoan Millepora complanata following the 2015-2016 ENSO in the Mexican Caribbean
Source: PeerJ. 2023 Jan 18;11:e14626. doi: 10.7717/peerj.14626 (PMC9864129; doi:10.7717/peerj.14626)
Supplement: Supplemental Information 4 — Based on the number of sequences matching species, M. complanata metatranscriptome mainly contained putative homologs to Symbiodinium microadriaticum and Hydra vulgaris. [file peerj-11-14626-s004.docx]

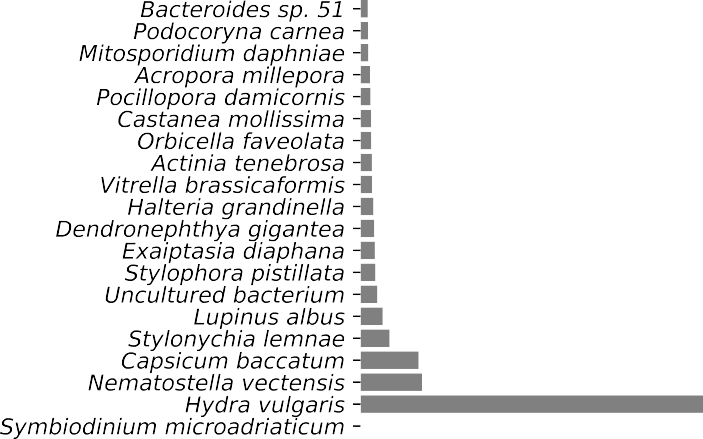

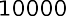

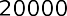

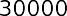

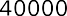

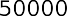


**Supplemental Figure S4.** Top-20 hit species from *M. complanata* metatranscriptome. Based on the number of sequences matching species, *M. complanata* metatranscriptome mainly contained putative homologs to *Symbiodinium miroadriaticum* and *Hydra vulgaris.*
