# Supplementary material for: Transcriptomic differences between bleached and unbleached hydrozoan Millepora complanata following the 2015-2016 ENSO in the Mexican Caribbean
Source: PeerJ. 2023 Jan 18;11:e14626. doi: 10.7717/peerj.14626 (PMC9864129; doi:10.7717/peerj.14626)
Supplement: Supplemental Information 5 — Right panel: Gene ontology terms across Biological Process (BP), Cellular Component (CC), and Molecular Function (MF) sub-ontologies under unbleached and bleached conditions. Left panel: GO terms with significant gene number differences (p < 0.05). [file peerj-11-14626-s005.docx]

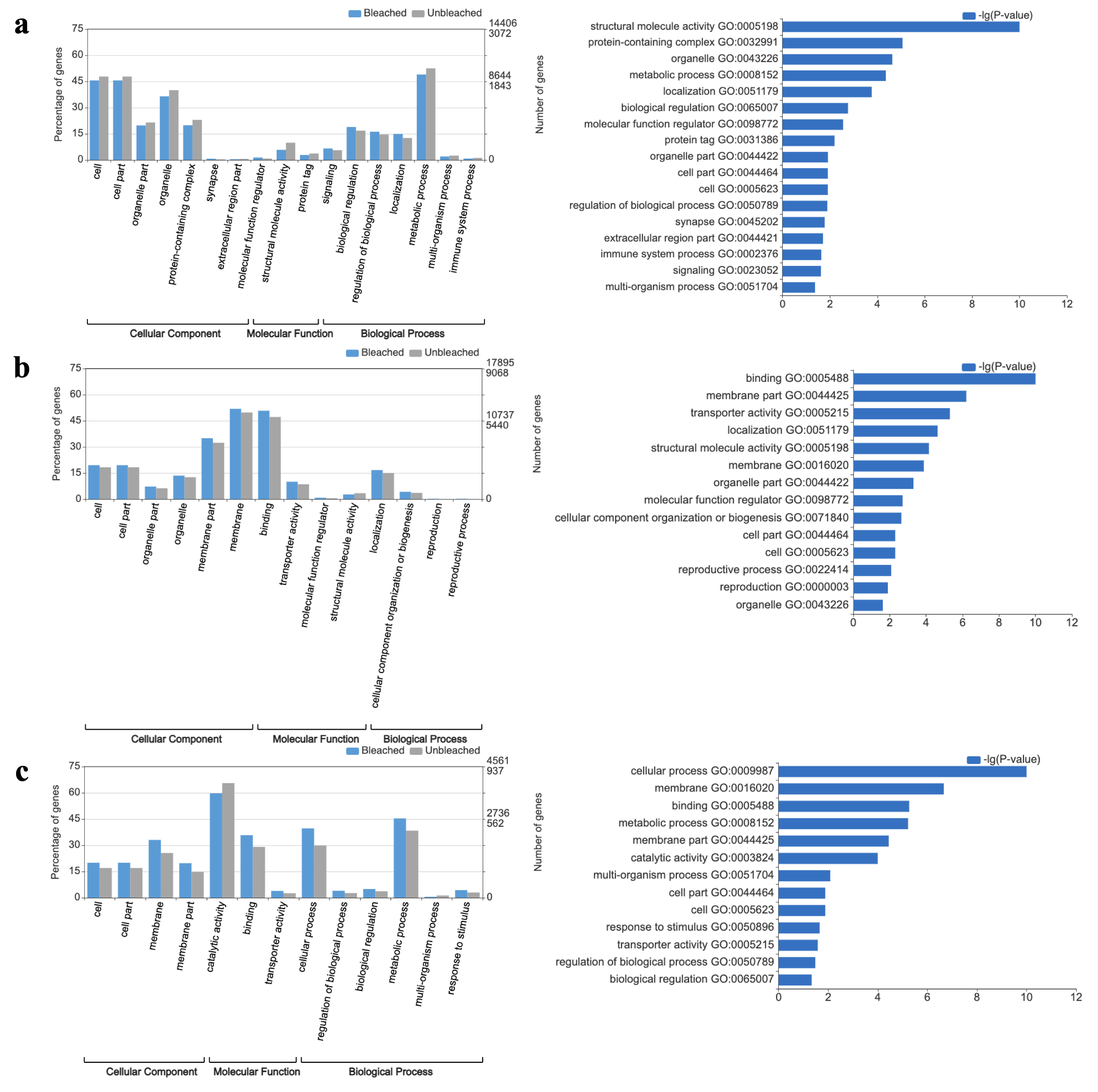


**Supplemental Figure S5.** Gene ontology terms for a) host, b) symbiont, and c) microbiome subsets. Right panel: Gene ontology terms across Biological Process (BP), Cellular Component (CC), and Molecular Function (MF) sub-ontologies under unbleached and bleached conditions. Left panel: GO terms with significant gene number differences (p<0.05).
