# Supplementary material for: Transcriptomic differences between bleached and unbleached hydrozoan Millepora complanata following the 2015-2016 ENSO in the Mexican Caribbean
Source: PeerJ. 2023 Jan 18;11:e14626. doi: 10.7717/peerj.14626 (PMC9864129; doi:10.7717/peerj.14626)
Supplement: Supplemental Information 7 — Overall, 147 KEGG pathways were retrieved for (A) unbleached and (B) bleached M. complanata. [file peerj-11-14626-s007.docx]

**a b**


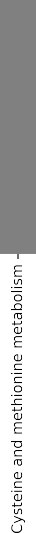

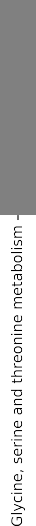

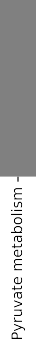

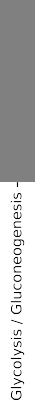

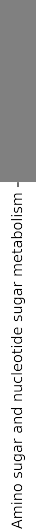

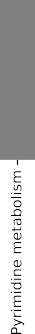

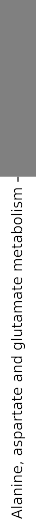

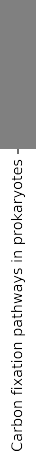

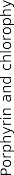

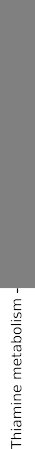

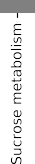

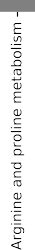

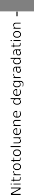

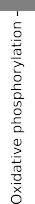

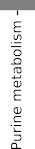

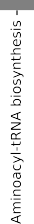

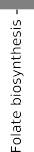

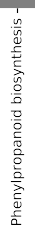

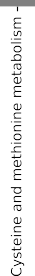

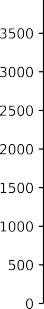


Number of sequences

Number of sequences

**Supplemental Figure S6.** The most represented metabolic pathways (based on the number of sequences per pathway) detected in the *M. complanata* holobiont. Overall, 147 KEGG pathways were retrieved for a) unbleached and b) bleached *M. complanata*.
