# Supplementary material for: Transcriptomic differences between bleached and unbleached hydrozoan Millepora complanata following the 2015-2016 ENSO in the Mexican Caribbean
Source: PeerJ. 2023 Jan 18;11:e14626. doi: 10.7717/peerj.14626 (PMC9864129; doi:10.7717/peerj.14626)
Supplement: Supplemental Information 8 [file peerj-11-14626-s008.docx]

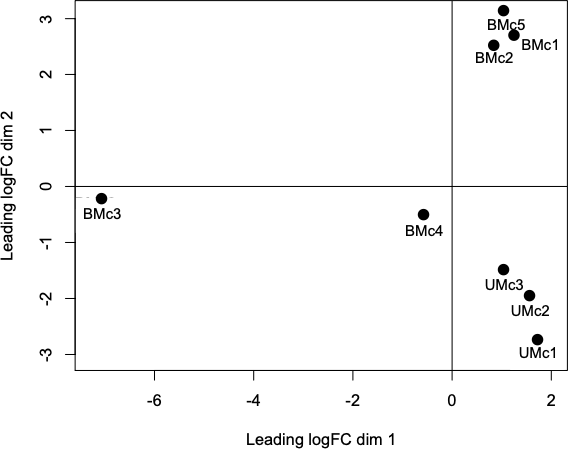


**Supplemental Figure S7.** Multidimensional Scaling analysis (simplified PCoA) showing the multivariate variation among unbleached and bleached *M. complanata* samples (UMc and BMc, respectively).
