# Supplementary material for: Transcriptomic differences between bleached and unbleached hydrozoan Millepora complanata following the 2015-2016 ENSO in the Mexican Caribbean
Source: PeerJ. 2023 Jan 18;11:e14626. doi: 10.7717/peerj.14626 (PMC9864129; doi:10.7717/peerj.14626)
Supplement: Supplemental Information 9 — Asterisks (*) indicate statistical significance (p < 0.05). Calculated p-values for expression levels: 0.0175, 0.0237, 0.0237, 0.0033, and 0.0039 for SOD, MET, MIO, HSP, and CCH, respectively. Control) S′adenosyl-l-methionine (SAM). (B) Comparison of gene expression trend by RNA-Seq and semi-quantitative RT-PCR. [file peerj-11-14626-s009.docx]

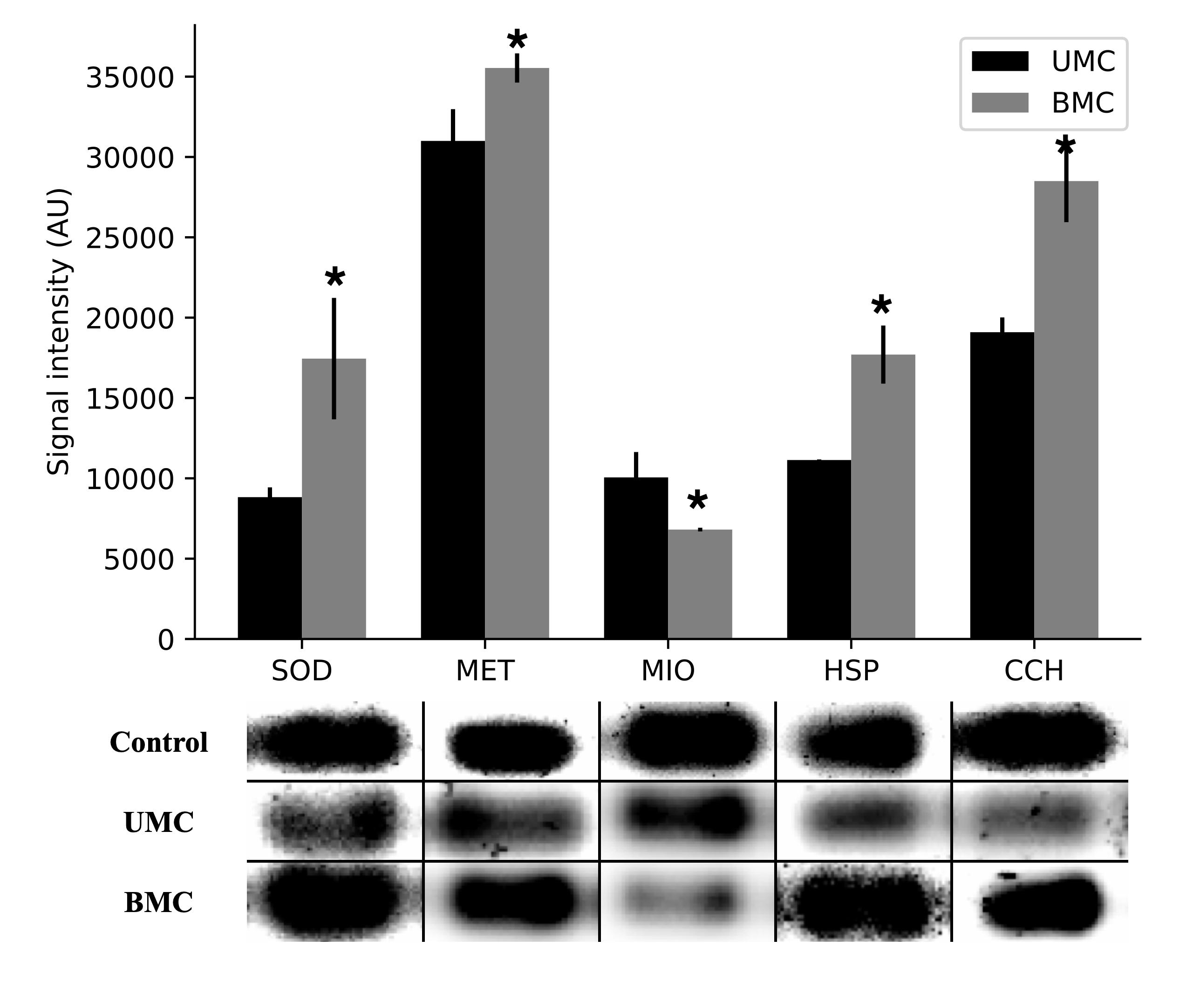
**a**

| **b** | **Sequence identifier** | **Accession** | **Description** | **E-value** | **Expression trend RNA- Seq** | **Fold- change** | **Expression trend semi- quantitative PCR** | **Mean signal intensity (UA)** |
| --- | --- | --- | --- | --- | --- | --- | --- | --- |
|  | Mcom_162778 | SODC_BOMMO | Superoxide dismutase | 3.67142E-08 | **U** | 349.58 | **U** | 17,460.0 |
|  | Mcom_309 | NAS6_CAEEL | Zinc metalloproteinase nas-6 | 2.11041E-17 | **U** | 340.09 | **U** | 35,593.0 |
|  | Mcom_289194 | MYS_PODCA | Myosin heavy chain | 4.08753E-78 | **D** | -147.19 | **D** | 10,058.0 |
|  | Mcom_5556 | CH10_SCHJA | 10 kDa heat shock protein, mitochondrial | 3.44982E-33 | **U** | 123.87 | **U** | 17,702 |
|  | Mcom_40833 | CACB2_RABIT | Voltage-dependent L-type calcium channel subunit beta-2 | 1.86888E-139 | **U** | 296.93 | **U** | 28,500.0 |
|  | U) Up- regulated |  |  |  |  |  |  |  |
|  | D) Down- regulated |  |  |  |  |  |  |  |

**Supplemental Figure S8.** a) Relative expression of five randomly selected DEGs. SOD) superoxide dismutase, MET) zinc-metalloproteinase, MIO) myosin heavy chain, HSP) 10 kDa heat shock protein, and CCH) voltage-dependent L-type calcium channel subunit beta-2. Validations were carried out with semi-quantitative PCR. UMC) Unbleached *M. complanata.* BMC) Bleached *M. complanata.* Asterisks (*) indicate statistical significance (p < 0.05). Calculated p-values for expression levels: 0.0175, 0.0237, 0.0237, 0.0033, and 0.0039 for SOD, MET, MIO, HSP, and CCH, respectively. Control) *S*′adenosyl-l-methionine (SAM). b) Comparison of gene expression trend by RNA-Seq and semi-quantitative RT-PCR.
