# Supplementary material for: Transcriptomic differences between bleached and unbleached hydrozoan Millepora complanata following the 2015-2016 ENSO in the Mexican Caribbean
Source: PeerJ. 2023 Jan 18;11:e14626. doi: 10.7717/peerj.14626 (PMC9864129; doi:10.7717/peerj.14626)
Supplement: Supplemental Information 10 [file peerj-11-14626-s010.docx]

**Supplemental Table S1.** Primers employed for Symbiodiniaceae identification using PCR.

# Symbiodiniaceae genus

**Target Forward sequence Reverse sequence**

*Symbiodinium* ITS2 CCTCTTGGACCTTCCACA

AC

*Breviolum* LSU-28S GTCTTTGTGAGCCTTGAG

C

*Cladocopium* ITS1 AAGGAGAAGTCGTAACA

AGGTTTCC

*Durisdinium* ITS1 AAGGAGAAGTCGTAACA

AGGTTTCC

GCATGCAGCAACACTGCT C

GCACACTAACAAGTGTAC CATG

AAGCATCCCTCACAGCCA AA

CACCGTAGTGGTTCACGT GTAATAG
