# Supplementary material for: Transcriptomic differences between bleached and unbleached hydrozoan Millepora complanata following the 2015-2016 ENSO in the Mexican Caribbean
Source: PeerJ. 2023 Jan 18;11:e14626. doi: 10.7717/peerj.14626 (PMC9864129; doi:10.7717/peerj.14626)
Supplement: Supplemental Information 11 [file peerj-11-14626-s011.docx]

**Supplemental Table S2.** Primers used for DEGs validation with semi-quantitative PCR.

# Description Forward sequence Reverse sequence

Superoxide dismutase GGCGTGGATATTAAAAA CAG

Zinc metalloproteinase nas-6 CAGAATGGATGTCATTG

TGA

Myosin heavy chain TAGCTCTTTGTCTGGCTT TC

ATGGACCGTACATTGGT AAG

TGTTCTCTTCGACGTAA CCT

GCAAGACGCTACAGAA AAAT

10 kDa heat shock protein, mitochondrial

Voltage-dependent L-type calcium channel subunit beta-2

TACCAGTTTCCGTAGCA GTT

CTCGTAGCTGCTGAAAA GTT

TCTGCGTTTGTAATTCAC TG

GTATTGACGTAGCTCCC AAG
