# Supplementary material for: Transcriptomic differences between bleached and unbleached hydrozoan Millepora complanata following the 2015-2016 ENSO in the Mexican Caribbean
Source: PeerJ. 2023 Jan 18;11:e14626. doi: 10.7717/peerj.14626 (PMC9864129; doi:10.7717/peerj.14626)
Supplement: Supplemental Information 12 [file peerj-11-14626-s012.docx]

**Supplemental Table S3.** Annotated DEGs by the effect of thermal stress in bleached *M. complanata*.

| **Sequence identifier** | **Accession** | **Description** | **E-value** | **#GO**  **IDs** |  | **Expression** | **Fold-change** | **LogFold- change** |
| --- | --- | --- | --- | --- | --- | --- | --- | --- |
| Mcom_334265 | SAC97892.1 | Uncharacterized protein ORF91 (chloroplast) | 1.45317E-17 | 1 | | D | 1276.7240890 | 10.3182310 |
| Mcom_340624 | XP_020620022.1 | Cytochrome c | 3.25655E-60 | 8 | | U | 44100.200796 | 15.4284976 |
| Mcom_21730 | AER54540.1 | Cytochrome c oxidase subunit 2 | 3.17665E-107 | 7 | | U | 182.73755289 | 7.51362933 |
| Mcom_11861 | XP_002153881.1 | Cytochrome c oxidase subunit 6A | 5.06327E-18 | 3 | | U | 1262.4146378 | 10.3019701 |
| Mcom_368787 | XP_002160547.1 | NADH dehydrogenase | 3.85075E-114 | 10 | | U | 430.47354774 | 8.74978077 |
| Mcom_375232 | XP_002159185.1 | Sulfite oxidase, mitochondrial | 3.58009E-162 | 13 | | U | 147.27151996 | 7.20233465 |
| Mcom_379104 | XP_004207238.1 | Metaxin-1 | 2.07999E-41 | 1 | | U | 350.83160062 | 8.45463489 |
| Mcom_224758 | XP_006129875.1 | ATP synthase lipid-binding protein, | 1.25444E-36 | 14 | | D | 88.758090799 | 6.47180673 |
|  |  | mitochondrial |  |  | |  |  |  |
| Mcom_340556 | XP_012555294.1 | NADP-dependent malic enzyme | 1.21439E-116 | 23 | | U | 279.55910326 | 8.12700951 |
| Mcom_80486 | XP_012564774.1 | Alanine aminotransferase 2 | 0.0 | 12 | | U | 695.43274667 | 9.44176719 |
| Mcom_14267 | XP_002155934.1 | Ethanolamine-phosphate cytidylyltransferase | 1.72585E-158 | 6 | | U | 521.54595559 | 9.02665057 |
| Mcom_5341 | EDO36122.1 | EH domain-containing protein 2 | 1.24308E-08 | 3 | | U | 463.42224139 | 8.85618347 |
| Mcom_333904 | XP_002158873.2 | Lysosome-associated membrane glycoprotein | 1.30311E-12 | 25 | | D | 69.453508788 | 6.11797567 |
|  |  | 1 |  |  | |  |  |  |
| Mcom_375516 | ACY74447.1 | Actin | 0.0 | 3 | | U | 302.06468295 | 8.23871370 |
| Mcom_340435 | XP_002160112.1 | Radixin 2438 | 0.0 | 63 | | U | 64.555725362 | 6.01247314 |
| Mcom_289194 | Q05000.1 | Myosin heavy chain | 4.08753E-78 | 27 | | D | 147.19053936 | 7.20154113 |
| Mcom_132637 | XP_012556891.1 | ATP-binding cassette sub-family A member 2 | 2.0417E-124 | 74 | | U | 286.27767189 | 8.16127134 |
| Mcom_40833 | ABD59026.1 | Voltage-dependent L-type calcium channel | 1.86888E-139 | 28 | | U | 296.93077351 | 8.21398280 |
|  |  | subunit beta-2 |  |  | |  |  |  |
| Mcom_41059 | XP_002160025.1 | Protein RER1 | 1.60237E-83 | 12 | | U | 277.64879416 | 8.11711732 |
| Mcom_369436 | XP_002154626.1 | Protein transport protein Sec61 subunit beta | 2.94603E-23 | 14 | | U | 59.844979899 | 5.90315832 |
| Mcom_376281 | XP_002164532.3 | Charged multivesicular body protein 7 | 6.37689E-31 | 7 | | U | 339.90713191 | 8.40899682 |
| Mcom_352094 | XP_002156778.1 | GTP-binding protein SAR1a | 3.81118E-97 | 16 | | U | 948.01404318 | 9.88876462 |
| Mcom_228382 | XP_006822187.1 | Vacuolar protein sorting-associated protein 11 | 2.9801E-60 | 28 | | D | 89.428491540 | 6.48266263 |
|  |  | homolog |  |  | |  |  |  |
| Mcom_29474 | XP_002160344.2 | General transcription factor IIF subunit 2 | 2.80607E-67 | 10 | | U | 294.82171345 | 8.20369897 |
| Mcom_96698 | XP_027048494.1 | Circadian locomoter output cycles protein | 2.54647E-62 | 55 | | U | 279.17234612 | 8.12501222 |
|  |  | kaput-like isoform X2 |  |  | |  |  |  |
| Mcom_63 | XP_002163509.2 | DNA-directed RNA polymerase II subunit | 1.5809E-118 | 24 | | U | 676.54639494 | 9.40204506 |
|  |  | RPB2 |  |  | |  |  |  |
| Mcom_368775 | XP_002157035.1 | Protein arginine N-methyltransferase 1 | 7.10301E-170 | 42 | | U | 127.66644451 | 6.99623557 |

| Mcom_132054 | XP_001629792.2 KAT8 | KAT8 regulatory NSL complex subunit 3 | 7.89375E-74 | 3 | U | 2374.6772941 | 11.2135157 |
| --- | --- | --- | --- | --- | --- | --- | --- |
| Mcom_97851 | XP_002156165.1 | ATP-dependent RNA helicase DDX18 | 3.80115E-103 | 14 | U | 329.69078307 | 8.36496974 |
| Mcom_164457 | PIK45035.1 | Putative transcription elongation factor B polypeptide 1 | 3.07025E-59 | 15 | U | 497.49518082 | 8.95853874 |
| Mcom_51 | XP_002167484.2 | Probable glucosamine 6-phosphate N- acetyltransferase | 4.68977E-34 | 12 | U | 325.06917307 | 8.34460293 |
| Mcom_286392 | CBJ32893.1 | Elongation factor 1-alpha | 0.0 | 28 | D | 70.341240303 | 6.13629886 |
| Mcom_29613 | XP_004210536.1 | 60S ribosomal protein L28 | 5.30798E-34 | 15 | U | 89.095766380 | 6.47728497 |
| Mcom_352275 | XP_002162844.1 | 60S ribosomal protein L15 | 4.53887E-94 | 5 | U | 13295.477543 | 13.6986479 |
| Mcom_361440 | XP_002167181.1 | 60S ribosomal protein L3 | 0.0 | 8 | U | 78.611866946 | 6.29667520 |
| Mcom_361745 | XP_027039507.1 | Probable 60S ribosomal protein L37-A | 9.86898E-35 | 13 | U | 72.757634323 | 6.18502673 |
| Mcom_368908 | XP_002154604.1 | 60S ribosomal protein L7a | 1.52604E-104 | 16 | U | 79.572781668 | 6.31420312 |
| Mcom_381948 | XP_002157788.1 | Elongation factor 2 | 3.87495E-76 | 31 | U | 63.722524019 | 5.99373150 |
| Mcom_50654 | XP_002167039.1 | Signal peptidase complex catalytic subunit SEC11C | 8.32792E-98 | 4 | D | 104.03825942 | 6.70097035 |
| Mcom_392361 | XP_019626223.1 | Translation machinery-associated protein 7 | 7.53134E-10 | 1 | U | 62.725145157 | 5.97097199 |
| Mcom_34105 | XP_004206204.1 | 40S ribosomal protein S30 | 1.9916E-59 | 11 | U | 91.496877005 | 6.51565059 |
| Mcom_14531 | XP_012560354.1 | 40S ribosomal protein S21 | 1.79431E-29 | 14 | U | 83.868696518 | 6.39006052 |
| Mcom_22386 | XP_002154455.2 | 60S acidic ribosomal protein P0 | 1.01138E-13 | 18 | D | 28.202222189 | 4.81773693 |
| Mcom_8964 | EPY87465.1 | 60S ribosomal protein L38 | 9.50835E-33 | 13 | U | 1381.6141492 | 10.4321390 |
| Mcom_14548 | XP_020631005.1 | 60S ribosomal protein L30 | 2.47305E-58 | 14 | U | 1153.4018302 | 10.1716795 |
| Mcom_5558 | XP_012554015.1 | 40S ribosomal protein S14 | 1.45918E-95 | 16 | U | 821.89701084 | 9.68281381 |
| Mcom_14699 | XP_002158762.1 | 40S ribosomal protein S11 | 1.03139E-76 | 8 | U | 160.76250818 | 7.32878718 |
| Mcom_11771 | XP_002158630.1 | 40S ribosomal protein S8 | 1.94285E-101 | 11 | U | 85.993542817 | 6.42615642 |
| Mcom_375472 | XP_001640983.2 | Beta-1,4-galactosyltransferase 1 | 3.17841E-81 | 46 | U | 285.98184904 | 8.15977977 |
| Mcom_185 | XP_012562496.1 | Ubiquitin-protein ligase E3B | 1.09805E-16 | 2 | U | 465.34321040 | 8.86215134 |
| Mcom_14309 | XP_020914076.2 | Ribitol-5-phosphate xylosyltransferase 1 | 3.20071E-51 | 5 | U | 345.26238178 | 8.43154934 |
| Mcom_29387 | XP_034757966.1 | Polyubiquitin | 0.0 | 24 | U | 3636.6904282 | 11.8284104 |
| Mcom_40901 | XP_012557742.1 | DNA replication licensing factor mcm2 | 0.0 | 38 | U | 313.34615734 | 8.29161349 |
| Mcom_21683 | XP_002163825.1 | DNA replication complex GINS protein PSF3 | 9.97508E-56 | 3 | U | 567.92591233 | 9.14955892 |
| Mcom_132706 | XP_002167421.3 | Casein kinase I isoform gamma-3 | 0.0 | 11 | U | 380.13961889 | 8.57038558 |
| Mcom_14149 | XP_012556253.1 | Mitogen-activated protein kinase kinase kinase 3 | 1.44241E-93 | 26 | U | 754.99098480 | 9.56031560 |
| Mcom_194638 | XP_012555833.1 | Centromere-associated protein E | 1.25655E-102 | 28 | U | 315.57400988 | 8.30183458 |
| Mcom_21673 | XP_012561340.1 | THO complex subunit 3 | 1.87029E-160 | 18 | U | 459.89598390 | 8.84516378 |

| Mcom_132261 | XP_002165482.2 | U5 small nuclear ribonucleoprotein 200 kDa | 0.0 | 18 | U | 345.32168082 | 8.43179710 |
| --- | --- | --- | --- | --- | --- | --- | --- |
|  |  | helicase |  |  |  |  |  |
| Mcom_40894 | XP_002164714.2 | Probable ATP-dependent RNA helicase | 0.0 | 41 | U | 317.59567972 | 8.31104747 |
|  |  | DDX6 |  |  |  |  |  |
| Mcom_162432 | XP_002161749.2 | Probable ATP-dependent RNA helicase | 0.0 | 14 | U | 1642.7803089 | 10.6819238 |
|  |  | DDX46 |  |  |  |  |  |
| Mcom_377629 | XP_002164119.1 | Serine/arginine-rich splicing factor 6 | 8.55599E-49 | 25 | U | 1069.8960433 | 10.0632549 |
| Mcom_309 | XP_034553585.1 | Zinc metalloproteinase nas-6 | 2.11041E-17 | 13 | U | 340.09204195 | 8.40978143 |
| Mcom_376960 | XP_002166019.1 | U11/U12 small nuclear ribonucleoprotein 35 | 6.05631E-43 | 9 | U | 72.879056743 | 6.18743238 |
|  |  | kDa protein |  |  |  |  |  |
| Mcom_162778 | JQ994220.1 | Superoxide dismutase | 3.67142E-08 | 6 | U | 349.58206831 | 8.44948737 |
| Mcom_368760 | XP_002160295.1 | Soma ferritin | 3.51062E-59 | 9 | U | 678.64708248 | 9.40651771 |
| Mcom_5390 | XP_018080893.1 | Hypoxia-inducible factor 1-alpha inhibitor | 2.26163E-107 | 25 | U | 324.30995202 | 8.34122948 |
| Mcom_40964 | XP_002155810.1 | Complement component 1 Q subcomponent- | 4.29566E-21 | 16 | U | 341.53278481 | 8.41588026 |
|  |  | binding protein, mitochondrial |  |  |  |  |  |
| Mcom_379896 | XP_027131091.1 | Cysteine-rich protein 1 | 6.1676E-29 | 19 | U | 53.319565259 | 5.73659311 |
| Mcom_11680 | XP_012560101.1 | Nucleolin 2-like isoform X1 | 6.57403E-20 | 11 | U | 1400.8493747 | 10.4520861 |
| Mcom_5556 | XP_002162621.1 | 10 kDa heat shock protein | 3.44982E-33 | 10 | U | 123.87118192 | 6.95269677 |
| Mcom_340704 | XP_002155592.2 | 12 kDa FK506-binding protein | 2.7987E-47 | 55 | U | 149.75229167 | 7.22643427 |
| Mcom_14432 | XP_002164619.2 | NAD(P)H-hydrate epimerase | 1.11441E-103 | 13 | U | 652.20844224 | 9.34918930 |
| Mcom_240920 | OBZ62712.1 | Hypothetical protein A0H81_15016 | 3.82223E-09 | 3 | D | 86.822688215 | 6.44000018 |
| U: Up-reulated  D: Down-regulated | | | | | | | |
